# Outcomes following reverse total shoulder arthroplasty vs operative fixation for proximal humerus fractures: a systematic review and meta-analysis

**DOI:** 10.1308/rcsann.2022.0120

**Published:** 2023-12-01

**Authors:** SM Heo, H Faulkner, VVG An, M Symes, H Nandapalan, B Sivakumar

**Affiliations:** ^1^ Hornsby Ku-ring-gai Hospital, Sydney, Australia; ^2^ Royal Prince Alfred Hospital, Sydney, Australia; ^3^ Royal North Shore Hospital, Sydney, Australia; ^4^ St George Hospital, Sydney, Australia; ^5^ Hawkesbury Hospital, Sydney, Australia

**Keywords:** Proximal humerus fracture, Reverse total shoulder arthroplasty, Open reduction and internal fixation, Clinical outcomes, Patient reported outcome measures

## Abstract

**Introduction:**

Proximal humerus fractures are common in the older population. A consensus on the optimal management of complex fractures requiring surgery has yet to be reached. A systematic review and meta-analysis was performed to compare clinical outcomes between reverse total shoulder arthroplasty (RTSA) and open reduction and internal fixation (ORIF).

**Methods:**

A systematic search of the literature was undertaken using the Medline^®^, PubMed, Embase™ and Cochrane Central Register of Controlled Trials databases. Prospective and retrospective studies comparing clinical and patient reported results as primary outcome measures were included in this review, with secondary outcome measures including complications and revision surgery. A meta-analysis was conducted.

**Results:**

A total of 326 patients from 5 studies were eligible for inclusion in this review. Superior Constant–Murley scores (mean difference [MD]: 13.4, 95% confidence interval [CI]: 6.2–20.6; *p*<0.001), Oxford shoulder scores (MD: 4.3, 95% CI: 1.2–7.4; *p*=0.007), simple shoulder test scores (MD: 0.95, 95% CI: 0.01–1.89; *p*=0.05) and DASH (Disabilities of the Arm, Shoulder and Hand) scores (MD: 5.1 [1 study], 95% CI: 2.1–8.1; *p*=0.034) were noted in patients receiving RTSA. Range of motion and revision surgery rates were also superior in this group.

**Conclusions:**

This study suggests that RTSA affords more favourable outcomes and lower revision rates than ORIF following proximal humerus fractures. Definitive conclusions are precluded, however, owing to small sample sizes and risk of bias in retrospective studies.

## Introduction

Proximal humerus fractures are common among older patients and have been increasing in incidence over the last three decades owing to an ageing population.^1–4^ Patients with simple undisplaced fractures may be treated satisfactorily without surgery but significant benefit can be gained with operative intervention for complex fractures in high functioning individuals.^5^

The two most common surgical options are open reduction and internal fixation (ORIF), and reverse total shoulder arthroplasty (RTSA), with the choice being dependent on several variables including fracture pattern, baseline patient demographics, and surgeon experience and preference. Although a probability model study published in 2020 predicted higher quality of life and greater long-term cost effectiveness with RTSA for proximal humeral fractures compared with ORIF,^6^ a consensus on the optimal management has yet to be reached. Consequently, the aim of this systematic review was to compare clinical outcomes between RTSA and ORIF for the management of proximal humerus fractures in the older population.

## Methods

A systematic review of the literature was conducted in March 2021 in accordance with the PRISMA (Preferred Reporting Items for Systematic Reviews and Meta-Analyses) guidelines,^7^ utilising the Ovid Medline^®^, PubMed, Embase™ and Cochrane Central Register of Controlled Trials databases. In order to maximise the sensitivity of the search strategy, various combinations of the terms ‘osteosynthesis’, ‘proximal humerus’, ‘fixation’, ‘reverse shoulder’, ‘shoulder replacement’, ‘shoulder arthroplasty’ and ‘fracture’ were used. After exclusion of duplicate records, abstracts were screened for relevance prior to undergoing full-text appraisal by two independent assessors. Inclusion of studies was decided by consensus, with any disagreements mediated by the senior author (BS). The reference lists of included studies were also reviewed for further articles for potential incorporation.

### Study inclusion and selection

Studies were included in this review if they were published in the English language, provided comparison between RTSA and ORIF in older patients following a proximal humerus fracture, and reported original data including at least one clinical outcome measure. Case reports, abstracts, conference presentations and studies reporting results following non-operative management or other surgical interventions were excluded. Data were extracted from the text, tables and figures of all included studies.

### Outcomes reporting

Primary outcome measures assessed comprised patient reported outcome measures (PROMs) including: American Shoulder and Elbow Surgeons (ASES) standardised shoulder assessment; Constant–Murley shoulder outcome score (CMS); Disabilities of the Arm, Shoulder and Hand (DASH) score; Oxford shoulder score (OSS); simple shoulder test (SST); and range of motion measurements. Secondary outcome measures comprised complications, including revision surgery and infection.

### Quality assessment

The quality of included studies was assessed using the *Journal of Bone and Joint Surgery* levels of evidence.^8^ Two researchers independently assessed the risk of bias with the risk of bias in non-randomised studies (ROBINS-I) tool.

### Statistical analysis

Statistical analysis was performed with RevMan 5.3 (Nordic Cochrane Centre, Copenhagen, Denmark). Outcome measures were combined using a random effects model. Mean difference with 95% confidence intervals (CIs) were used to summarise continuous outcomes and relative risk with 95% CIs were employed for dichotomous outcomes. An alpha of 0.05 was set for all outcomes.

## Results

**Figure 1:**
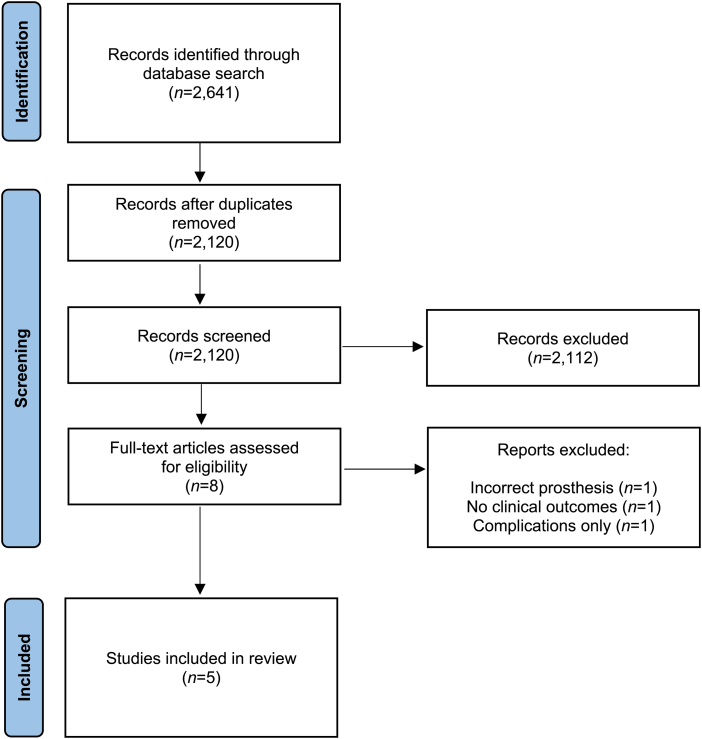
Flowchart of study selection

The electronic database search yielded 2,641 studies. Following removal of duplicates, 2,120 studies remained for title and abstract screening. Eight studies underwent full-text review. Three studies were excluded for not satisfying the inclusion criteria: one compared RTSA with humerus block, another analysed and compared cost effectiveness of the two procedures, and the last study reported exclusively on complications. Five articles were eligible for inclusion in the systematic review, with no additional references identified on full-text review (Figure 1). Four studies were observational in nature (level 3 evidence)^9–12^ whereas one was a randomised controlled trial (level 1).^13^

The final cohort comprised 326 patients, of whom 162 underwent RTSA and 164 received ORIF. A small but significant difference in the mean age of patients undergoing the different procedures was observed (RTSA 75.3 years vs ORIF 73.5 years, *p*=0.008). There was no statistical difference in the proportion of women undergoing either procedure (RTSA 88.9% vs ORIF 84.8%, *p*=0.28). The mean follow-up duration was 35.0 months, with a range of 10 months to 8 years.

In terms of PROMs, CMS was assessed in four studies, with the remaining outcome measures reported in two studies each. Complications were reported in all studies. Characteristics of the included studies are summarised in Table 1.

**Table 1:** Summary of the five included studies

Study	Level of evidence*	Mean follow-up duration	RTSA	ORIF	Outcome scores	Complications
Greiwe, 2020^9^ US	3	4.4 years	*n*=25	*n*=25	ASES, SST	Yes
Klug, 2020^10^ Germany	3	4.1 vs 3.2 years	*n*=30	*n*=30	CMS, OSS, ASES, DASH	Yes
Luciani, 2020^11^ Italy	3	2.8 vs 3.3 years	*n*=22	*n*=26	CMS, DASH	Yes
Giardella, 2017^12^ Italy	3	Unspecified	*n*=21	*n*=23	CMS, SST	Yes
Fraser, 2020,^13^ Norway	1	2 years	*n*=64	*n*=60	CMS, OSS	Yes
ASES = American Shoulder and Elbow Surgeons; CMS = Constant–Murley score; DASH = Disabilities of the Arm, Shoulder and Hand; ORIF = open reduction and internal fixation; OSS = Oxford shoulder score; RTSA = reverse total shoulder arthroplasty; SST = simple shoulder test						

*Journal of Bone and Joint Surgery levels of evidence^8^

The single randomised controlled trial (known as the Delta prosthesis–Philos plate [DelPhi] trial) consisted of 124 patients with displaced proximal humerus fractures corresponding to type B2 and C2 in the Orthopaedic Trauma Association/Arbeitsgemeinschaft für Osteosynthesefragen classification;^13^ 64 patients were randomised to RTSA with the remainder undergoing ORIF. Owing to a lack of standard deviation reporting, this study was excluded from meta-analysis. Data from the remaining four retrospective studies were pooled to allow a cohort of 202 patients: 98 patients were treated with RTSA and 104 with ORIF. Three studies classified fracture pattern using the Neer system, with distribution shown in Table 2.

**Table 2:** Fracture pattern by Neer classification in three observational studies of reverse total shoulder arthroplasty (RTSA) and open reduction and internal fixation (ORIF)^9–11^

	RTSA (*n*=77)	ORIF (*n*=81)
2-part	1 (1.3%)	9 (1.1%)
3-part	12 (15.6%)	30 (37.1%)
4-part	49 (63.6%)	38 (46.9%)
Head split	12 (15.6%)	2 (2.5%)
Fracture – dislocation	3 (3.9%)	2 (2.5%)

### Primary outcome measures

The DelPhi trial reported superior PROMs at two-year follow-up in patients undergoing RTSA compared with ORIF.^13^ A mean difference of 13.4 was noted in CMS (RTSA 68 vs ORIF 54.6, 95% CI: 6.2–20.6; *p*<0.001). Similarly, a mean increase of 4.3 was noted in OSS (RTSA 40.8 vs ORIF 36.5, 95% CI: 1.2–7.4; *p*=0.007). Patients undergoing RTSA also demonstrated superior flexion, abduction and external rotation at the end of the follow-up period (*p*<0.001). There was no significant difference in postoperative internal rotation between the two groups.

**Figure 2:**
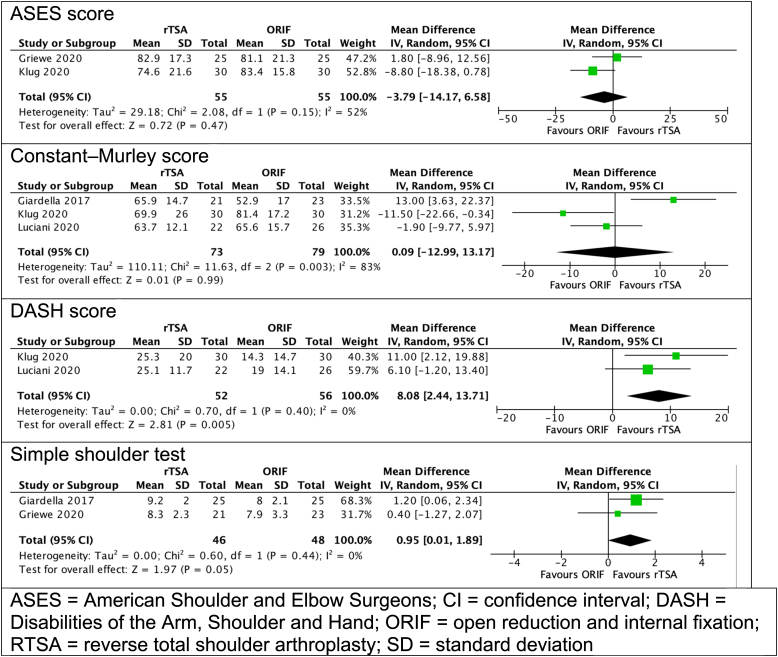
Patient reported outcome measures for patients undergoing RTSA and ORIF^9–12^

Among the four retrospective observational studies, heterogenous reporting of PROMs was noted, with meta-analysis performed on pooled scores where possible. Two studies reported SST scores, with a mean difference of 0.95 favouring RTSA patients (95% CI: 0.01–1.89, *p*=0.05). Patients undergoing RTSA also demonstrated a superior postoperative DASH score (mean difference: 8.08 [2 studies], 95% CI: 2.44–13.71; *p*=0.05) and OSS (mean difference: 5.1 [1 study]. 95% CI: 2.1–8.1; *p*=0.034). No significant difference was noted when considering CMS and ASES standardised shoulder assessment (Figure 2).

**Figure 3:**
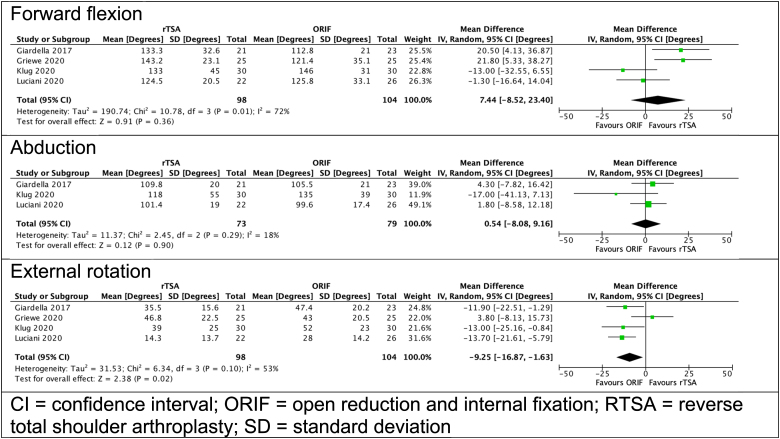
Range of motion scores for patients undergoing RTSA and ORIF^9–12^

In terms of range of motion, patients undergoing ORIF demonstrated superior external rotation (mean difference: 9.25°, 95% CI: 1.63–16.87°; *p*=0.020). No difference was noted in flexion or abduction (Figure 3). Owing to heterogeneous reporting of outcomes, internal rotation could not be compared.

### Secondary outcome measures

All five studies included in this systematic review reported postoperative complications. Owing to heterogeneity in complication reporting, it was not possible to perform a meta-analysis. The overall complication rate for the RTSA cohort was 22.8% compared with 29.9% for ORIF patients. The most common complications for those undergoing RTSA were scapular notching (*n*=13), and delayed union, malunion and non-union of the tuberosities (*n*=10); the most frequent complications following ORIF were hardware penetration (*n*=12) and avascular necrosis (*n*=12). Table 3 summarises the complications.

**Table 3:** Complications following reverse total shoulder arthroplasty (RTSA) and open reduction and internal fixation (ORIF)

Complication	RTSA	ORIF
Delayed union/malunion/non-union	10	6
Scapular notching	13	0
Avascular necrosis	0	12
Hardware penetration	0	12
Infection	4	2
Stiffness	0	6
Osteolysis/reabsorption	2	3
Nerve injury	3	1
Periprosthetic fracture	3	1
Loss of reduction	0	3
Instability	2	0
Subacromial impingement	0	2
Rotator cuff tear	0	1
**Total**	**37**	**49**

Patients undergoing RTSA also demonstrated lower rates of revision surgery (6 revisions at a rate of 4.3% compared with 22 revisions at a rate of 17.3%). The most common aetiologies for revision surgery following RTSA were periprosthetic fracture (*n*=2) and component change (*n*=2) while avascular necrosis (*n*=7) and hardware penetration (*n*=9) frequently led to reoperations following ORIF. Table 4 gives a summary of the aetiology leading to revision surgery.

**Table 4:** Reasons for revision surgery following reverse total shoulder arthroplasty (RTSA) and open reduction and internal fixation (ORIF)

Reason	RTSA	ORIF
Hardware penetration	0	9
Avascular necrosis	0	7
Loss of reduction	0	3
Component change	2	0
Infection	1	1
Periprosthetic fracture	2	0
Subacromial impingement	0	2
Instability	1	0
**Total**	**6**	**22**

### Quality assessment

**Figure 4:**
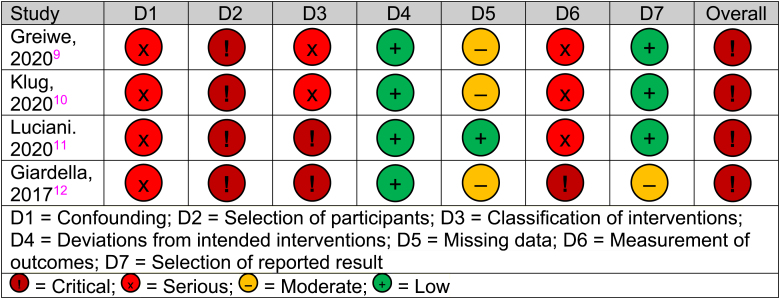
Risk of bias assessment according to the ROBINS-I tool

The DelPhi trial was deemed to be at low risk of bias when assessed by the revised Cochrane risk of bias tool for randomised trials. The remaining studies were assessed using the ROBINS-I tool and were deemed to have critical risk of bias (Figure 4).

## Discussion

Complex proximal humeral fractures in older patients are often treated by either RTSA or ORIF. Several previous systematic reviews have compared outcomes following non-operative management of proximal humeral fractures with various operative measures. However, this study is the first to compare RTSA with ORIF exclusively, with all included studies published after 2017, and to our knowledge, this has not been summarised previously. Patients undergoing RTSA following proximal humeral fractures demonstrated superior PROMs at a mean follow-up of 35.0 months. A small increase in external rotation was noted in ORIF patients. Higher rates of revision surgery were demonstrated following ORIF; this may be due to less osseous union needed for successful RTSA or the fact that no easy salvage operations exist for RTSA.

Conflicting results have been published previously when comparing RTSA with ORIF in the setting of assessing all therapeutic measures for proximal humeral fractures. Chen *et al* reported that RTSA afforded a significantly higher CMS as well as lower complication and revision rates than other treatment modalities, including ORIF, hemiarthroplasty, intramedullary nail and non-operative treatment.^14^ Conversely, Gupta *et al* observed an improved DASH score (*p*=0.014) and CMS (*p*=0.049) but higher revision rates (*p*<0.001) in patients undergoing ORIF when compared with RTSA.^15^ They concluded that RTSA may be the more effective intervention in this population given the large difference in revision rates. Based on the current study, owing to the improved outcome scores and lower revision rates, we would concur.

In patients with fractures of higher complexity, the DelPhi trial observed that RTSA patients reported significantly improved PROMs at the end of the follow-up period compared with ORIF patients.^13^ Unfortunately, further subgroup analysis based on fracture complexity could not be undertaken owing to insufficient data reporting in the remaining studies included for meta-analysis.^9–12^ Future evaluation of these modalities stratified by fracture complexity would be valuable.

Significant heterogeneity in the different PROMs utilised has been noted in the literature, limiting pooling of large cohorts.^16,17^ This was reflected in the current analysis; five different outcome measures were reported across the studies, with limited interpretation of cause and effect. A systematic review published in 2020 identified 22 PROMs used to report outcomes following proximal humerus fractures,^18^ with varying results published regarding score reliability and comparability.^19–21^ Although assessing the validity of each PROM used is outside the scope of this study, we advocate for the development of standard measures for pathology that are clinically accurate, cost effective and reproducible.

This systematic review has several limitations. Only one randomised controlled trial was available for inclusion and this study was not incorporated into the meta-analyses owing to a lack of sufficient data. The remaining retrospective observational studies lacked randomisation, leading to potential confounding and selection bias. As with all clinical studies, inconsistent and heterogeneous reporting of fracture classification and varying follow-up periods limit in-depth analysis.

## Conclusions

When performing surgery for proximal humerus fractures in older individuals, the treating clinician must consider the patient’s premorbid function, fracture pattern, bone quality and prognosis. The results of this review suggest that RTSA may be a superior alternative to ORIF, with improved PROMs and lower revision rates. However, further larger scale prospective studies, directly comparing these interventions and subdividing by fracture pattern, are required to confirm these findings.
